# Incidence, outcomes and risk factors of rebound neurological deterioration after initial improvement following intravenous thrombolysis: a retrospective cohort study

**DOI:** 10.3389/fnagi.2026.1706795

**Published:** 2026-03-27

**Authors:** Jinghan Hu, Lan Hong, Yifeng Ling, Siyuan Li, Xinru Wang, Anqi Zhang, Nan Cao, Qiang Dong, Zhijiao He, Xin Cheng

**Affiliations:** 1 Department of Neurology, National Center for Neurological Disorders, Huashan Hospital, Fudan University, Shanghai, China; 2 Department of Neurology, People’s Hospital of Wenshan Prefecture, Wenshan, Yunnan, China; 3 School of Medicine, Kunming University of Science and Technology, Kunming, Yunnan, China; 4 State Key Laboratory of Medical Neurobiology and MOE Frontiers Center for Brain Science, Fudan University, Shanghai, China

**Keywords:** acute ischemic stroke, early neurological improvement, intravenous thrombolysis, large vessel occlusion, rebound neurological deterioration

## Abstract

**Introduction:**

In clinical practice, a substantial number of acute ischemic stroke (AIS) patients experience neurological deterioration following by initial early neurological improvement (ENI) after intravenous thrombolysis (IVT), a pattern we define as rebound neurological deterioration (REND). However, the incidence and clinical implications of REND remain unclear.

**Methods:**

This retrospective study included AIS patients who achieved ENI, defined as ≥ 4-point reduction of National Institutes of Health Stroke Scale (NIHSS) score from baseline to 2 h after IVT. REND was defined as a ≥ 2-point increase in NIHSS score within 24 h from last known well, occurring after ENI and in the absence of symptomatic intracranial hemorrhage. The association between REND and functional outcomes was assessed using multivariable logistic regression models and the independent risk factors of REND were identified through backward stepwise selection.

**Results:**

A total of 1,025 AIS patients were screened in this retrospective study and 277 patients experienced ENI following IVT were included in the analysis. REND occurred in 19.9% of patients, which was negatively associated with favorable functional outcomes [adjusted OR (95%CI): 0.076 (0.031, 0.186), *P* < 0.001]. In stepwise logistic regression analysis, younger age [adjusted OR (95%CI): 0.966 (0.939, 0.994), *P* = 0.017], history of hypertension [adjusted OR (95%CI): 2.728 (1.165, 6.390), *P* = 0.021], and presence of large vessel occlusion/severe stenosis [adjusted OR (95%CI): 2.159 (1.076, 4.333), *P* = 0.030] were independent risk factors of REND.

**Conclusion:**

REND occurred in approximately one-fifth of AIS patients with ENI after IVT and was strongly associated with poor functional outcomes. Younger age, history of hypertension, and large vessel occlusion or severe stenosis were independent risk factors of REND.

## Introduction

Intravenous thrombolysis (IVT) remains a standard reperfusion therapy for patients with acute ischemic stroke (AIS) presenting within 4.5 h of symptom onset (Emberson et al., 2014), and is also recommended for selected patients with unknown or extended time windows based on computed tomography perfusion (CTP) or magnetic resonance imaging (MRI) (Thomalla et al., 2020; Xiong et al., 2024). Although IVT effectively improves functional outcomes in AIS patients, its therapeutic response varies substantially among individuals. Previous studies have reported that approximately 30∼40% patients achieved rapid neurological improvement within 2 h after IVT, which is associated with 90-day favorable functional outcomes (Broocks et al., 2023; Hemmen et al., 2011). In contrast, early neurological deterioration (END), typically defined as a significant worsening of neurological status within 24 h following IVT, has been consistently recognized as a strong predictor of poor prognosis (Che et al., 2022; Simonsen et al., 2016; Werring et al., 2025).

Most existing studies have focused on END following IVT, given its rapid progression and the need for urgent intervention (Liu et al., 2023; Sabir Rashid et al., 2022; Zhou et al., 2024). However, a distinct subgroup of patients who initially present early neurological improvement (ENI) but subsequently experience deterioration remains under explored in clinical practice. We define this “improvement-then-deterioration” trajectory as rebound neurological deterioration (REND), which may lead to reduced monitoring, delayed intervention, and ultimately, unfavorable functional outcomes.

REND has not been systematically investigated in previous studies and its pathophysiological mechanism, as well as prognostic significance remain poorly understood. Therefore, this study aimed to (1) determine the incidence and functional outcomes of REND following IVT, and (2) identify clinical and imaging risk factors of REND.

## Materials and methods

### Patient selection and clinical parameters

This retrospective observational study screened AIS patients who received IVT between September 2013 and September 2024 at the Department of Neurology, Huashan Hospital, Fudan University. This study specifically aimed to investigate neurological deterioration among patients who initially achieved ENI after IVT, so patients were included if they: (1) received IVT within 24 h from last known well, including those who subsequently underwent bridging endovascular treatment (EVT); (2) presented ENI, defined as ≥ 4-point reduction of National Institutes of Health Stroke Scale (NIHSS) score from baseline to 2 h after IVT. Exclusion criteria were (1) missing NIHSS scores records at either 2 or 24 h after receiving IVT; (2) presenting symptomatic intracranial hemorrhage (sICH) within 24 h. sICH was defined as any intracranial hemorrhage accompanied by a ≥ 4-point increase in NIHSS score from baseline, according to the European Cooperative Acute Stroke Study II (ECASS II) criteria.

REND was assessed as secondary neurological worsening after ENI. To evaluate the prognostic impact of different degrees of neurological worsening, we proposed two definitions of REND based on increased NIHSS score:

(1) REND was defined as an increase of 2 or more points in the NIHSS score within 24 h from the initiation of IVT, following ENI, with the absence of sICH.

(2) Substantial REND was further defined as an NIHSS increase of 4 or more points, using the same criteria.

The 3-month modified Rankin Scale (mRS) was used to assess functional disability and was evaluated through a structured telephone interview conducted by trained neurologists who were blinded to clinical data. favorable functional outcome was defined as mRS score of 0–1 at 3 months.

### Imaging analysis

CT perfusion (CTP) scanning was selectively performed according to clinical necessity. CTP imaging was post-processed by F-STROKE (Neuroblam, Co., Ltd., Shanghai, China). Previously validated thresholds were applied to measure the volume of hypoperfusion lesion [time to maximum > 6 s (Tmax > 6 s)] and core volume [relative cerebral blood flow (rCBF) < 30%].

Reperfusion was defined as a ≥ 50% reduction in hypoperfusion lesion volume on follow-up CTP compared to baseline in patients with large vessel occlusion (LVO) or severe stenosis, who underwent both baseline and repeated CTP within 24 h.

### Statistical analysis

Statistical Analysis was performed on SPSS (version 27.0, IBM Corp., Armonk, NY, United States). Figures were drawn with Adobe Illustrator 2021. Continuous variables were presented as mean and standard deviation (SD) if they were normally distributed, while continuous variables with skewed distribution were described as the median and interquartile range (IQR). Categorical variables were depicted as numbers and percentages. Differences in demographic, clinical, and imaging variables between patients with and without REND were compared using Student’s *t*-test or Wilcoxon rank-sum test for continuous variables, and chi-square test or Fisher’s exact test for categorical variables.

Variables with *p* < 0.1 on univariable analysis or of clinical relevance were entered into multivariable logistic regression models to explore the association between REND and favorable functional outcome. Backward stepwise selection was applied to identify independent risk factors of REND. The odds ratio (OR) and 95% confidence interval (CI) of the stepwise regression models were presented. A two-tailed *p*-value < 0.05 was considered statistically significant.

## Results

**FIGURE 1:**
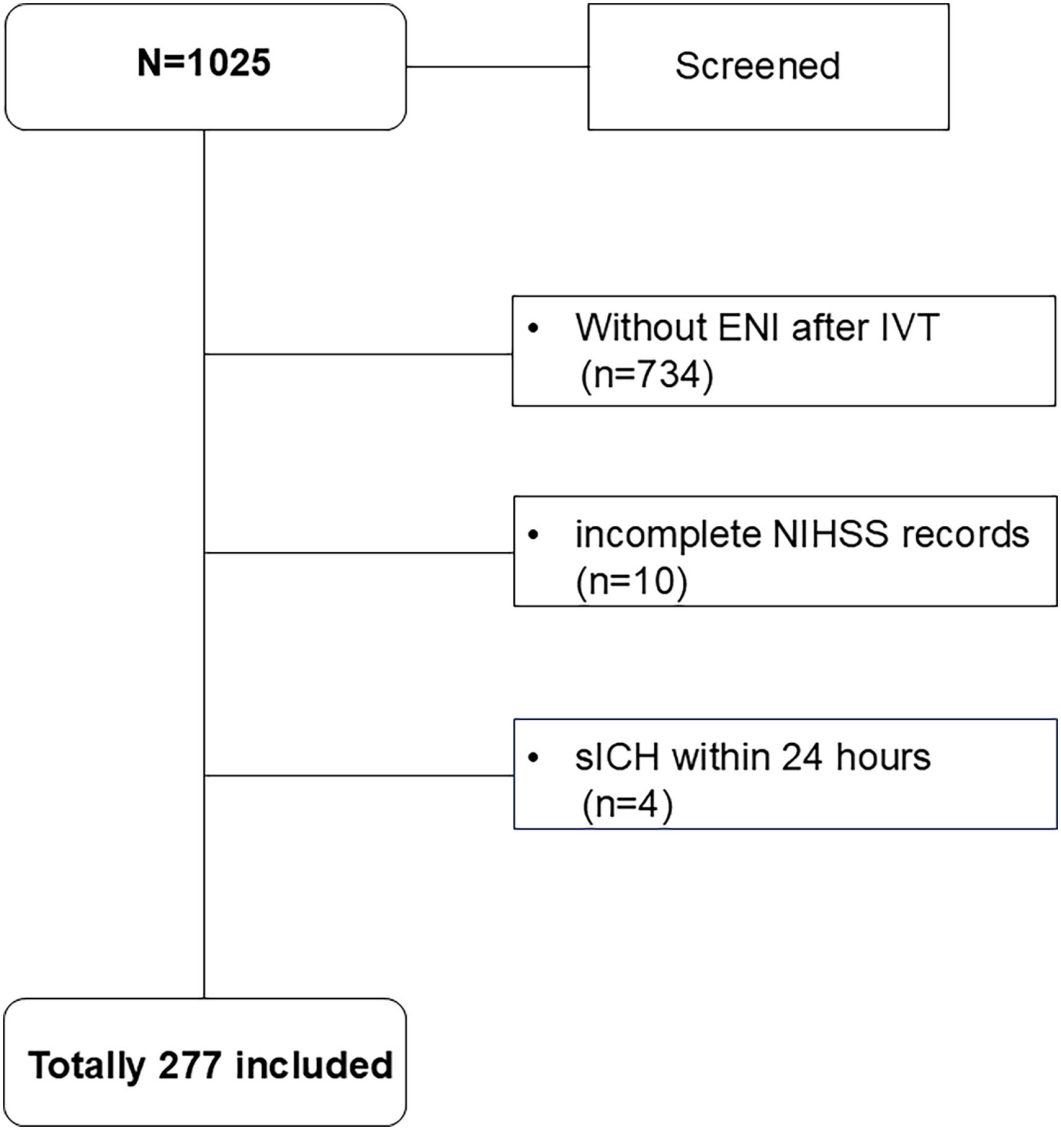
Flow-chart of patient selection. ENI, Early neurological improvement; NIHSS, National Institutes of Health Stroke Scale; sICH, symptomatic intracerebral hemorrhage.

Between September 2013 and September 2024, a total of 1,025 AIS patients who presented within 24 h of last known well received IVT and 277 patients were included in the final analysis (Figure 1).

The median (IQR) age of the study cohort was 68 (59, 76) years, and the median (IQR) NIHSS at admission was 11 (7, 15). The proportion of patients who received EVT in our study was 13.4% (37/277). Baseline demographic, clinical, and imaging characteristics of the study population were summarized in Table 1. In this cohort, REND was observed in 19.9% (55/277) of patients, and 13.0% (36/277) met the criteria for substantial REND. Vessel occlusion or severe stenosis was identified in 130 patients, among whom 57 completed repeated CTP within 24 h. The overall reperfusion rate in this subgroup was 73.7% (42/57).

**TABLE 1:** Demographic, imaging, and clinical characteristics of patients included.

Factors	All patients (*n* = 277)	Without REND (*n* = 222)	REND (*n* = 55)	*P*-value
Demographics				
Male sex, n (%)	199 (71.8%)	162 (73.0%)	37 (67.3%)	0.406
Age, years, median (IQR)	68 (59, 76)	69 (60, 76)	64 (55, 73)	0.031
Medical history				
Smoking, n (%)	122 (44.0%)	100 (45.5%)	22 (40%)	0.546
Hypertension, n (%)	184 (66.4%)	140 (63.1%)	44 (80.8%)	0.025
Diabetes mellitus, n (%)	69 (24.9%)	54 (24.3%)	15 (27.3%)	0.728
Hyperlipidemia, n (%)	37 (13.4%)	30 (13.5%)	7 (12.7%)	1.000
Stroke, n (%)	55 (19.9%)	44 (19.8%)	11 (20%)	1.000
Atrial fibrillation, n (%)	58 (20.9%)	45 (20.3%)	13 (23.6%)	0.712
Clinical features				
Admission NIHSS, median (IQR)	11 (7, 15)	10 (7, 16)	11 (7, 14)	0.925
NIHSS_2 h,median (IQR)	3 (1, 6)	3 (1, 7)	3 (1, 5)	0.593
NIHSS_24 h, median (IQR)	2 (1, 7)	2 (0, 4)	8 (5, 13)	< 0.001
OTD, min, median (IQR)	94 (54,150)	92 (51, 149)	104 (69, 157)	0.920
OTT, min, median (IQR)	155 (102, 212)	154 (100, 212)	160 (108, 220)	0.329
Admission SBP, mmHg, median (IQR)	148 (133, 171)	148 (133, 166)	153 (133, 171)	0.257
Admission DBP, mmHg, median (IQR)	84 (76, 93)	83 (75, 92)	89 (79, 98)	0.041
Plasma glucose, mmoL/L, median (IQR)	6.9 (5.7, 9.2)	6.9 (5.8, 9.2)	6.8 (5.6, 8.2)	0.656
Baseline NLR, median (IQR)	3.34 (2.12, 5.69)	3.17 (1.93, 5.32)	4.38 (2.91, 8.01)	0.004
Baseline fibrinogen, g/L, median (IQR)	2.7 (2.2, 3.3)	2.7 (2.2, 3.3)	2.9 (2.6, 3.7)	0.007
Baseline DDI, mg/L FEU, median (IQR)	0.99 (0.37, 2.57)	0.62 (0.32, 1.65)	1.52 (0.42, 2.83)	0.015
EVT, n (%)	37 (13.4%)	27 (12.2%)	10 (18.2%)	0.240
Vessel occlusion or severe stenosis, n (%)	130 (46.9%)	96 (43.2%)	34 (61.8%)	0.016
Site, n (%)				0.032
ICA	29 (10.5%)	19 (8.6%)	10 (18.2%)	
Tandem	2 (0.7%)	2 (0.9%)	0 (0%)	
MCA	78 (28.2%)	60 (27%)	18 (32.7%)	
ACA	6 (2.2%)	6 (2.7%)	0 (0%)	
PCA	2 (0.7%)	2 (0.9%)	0 (0%)	
BA	12 (4.3%)	7 (3.2%)	5 (9.1%)	
VA	1 (0.4%)	0 (0%)	1 (1.8%)	
Brain perfusion imaging features				
Reperfusion, n (%) *	42 (73.7%)	36 (83.7%)	6 (42.9%)	0.005
TOAST, n (%)				0.961
Large artery atherosclerosis	94 (33.9%)	74 (33.3%)	20 (36.4%)	
Cardioembolism	62 (22.4%)	51 (23%)	11 (20%)	
Small vessel occlusion	56 (20.2%)	45 (23.3%)	11 (20%)	
Other/undetermined cause	65 (23.5%)	52 (23.4%)	13 (23.6%)	
Prognosis				
Favorable functional outcomes, n (%)	155 (56.0%)	144 (64.9%)	11 (20%)	< 0.001

REND, rebound neurological deterioration; NIHSS, National Institutes of Health Stroke Scale; OTD, onset to door; OTT, onset to treatment; SBP, systolic blood pressure; DBP, diastolic blood pressure; NLR, neutrophil-lymphocyte ratio; DDI, D-dimer; EVT, endovascular treatment; ICA, internal carotid artery; MCA, middle cerebral artery; ACA, anterior cerebral artery; PCA, posterior cerebral artery; BA, basilar artery; VA, vertebral artery.

*Among 57 patients who completed both baseline and follow-up CTP imaging, 14 developed REND.

Compared to patients without REND, patients with REND were younger (median age: 64 vs. 69 years, *P* = 0.031), and more likely to have a history of hypertension (80.8% vs. 63.1%, *P* = 0.025), as well as vessel occlusion or severe stenosis (61.8% vs. 43.2%, *P* = 0.016). Inflammatory and coagulation markers also differed between groups: REND patients showed higher neutrophil-to-lymphocyte ratio (NLR) (4.38 vs. 3.17, *P* = 0.004), fibrinogen (2.9 vs. 2.7 g/L, *P* = 0.007), and D-dimer level (1.52 vs. 0.62 mg/L FEU, *P* = 0.015) (Table 1).

**FIGURE 2:**
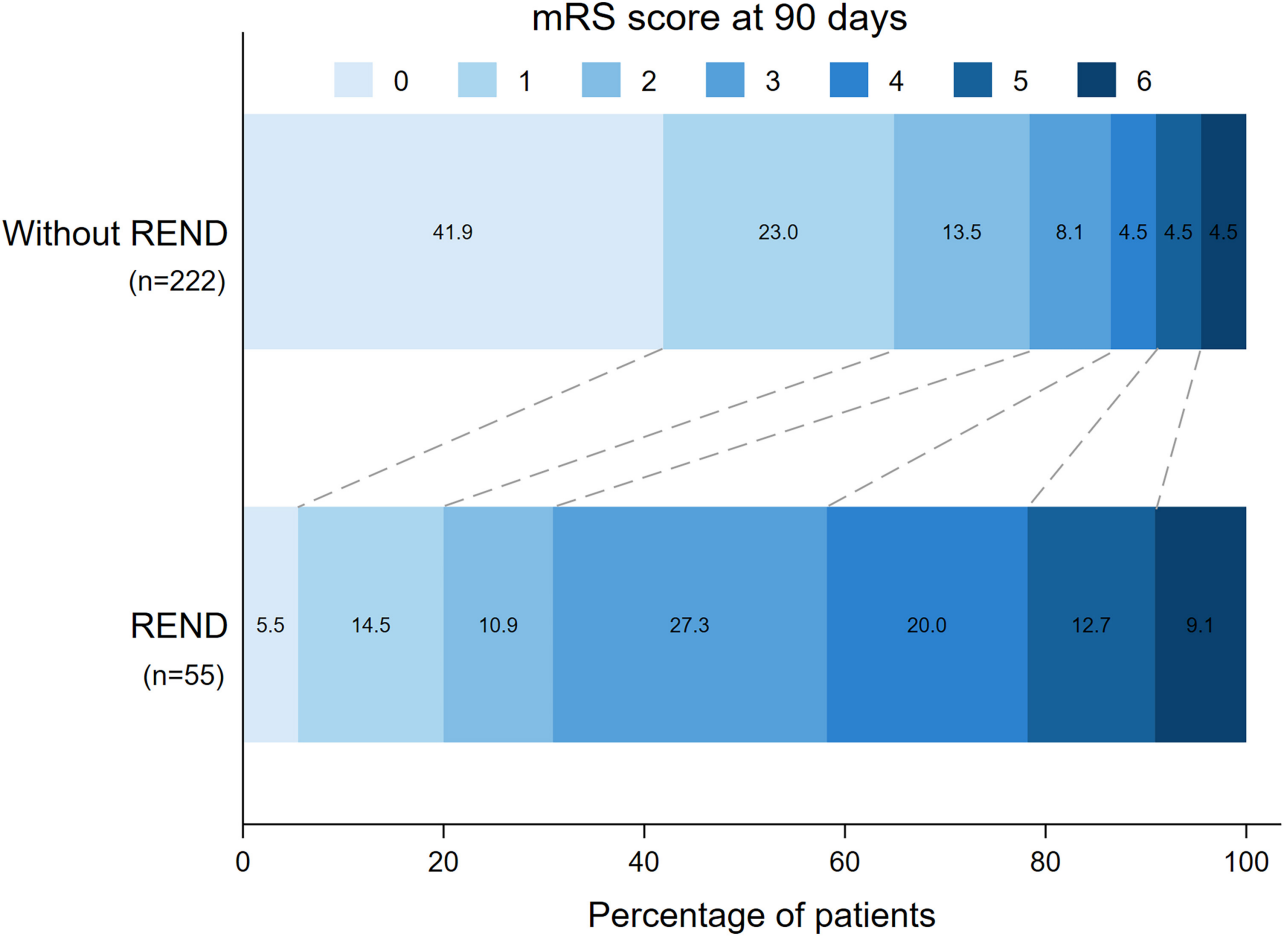
Distribution of modified Rankin scale scores at 90 days in patients with and without REND. mRS, modified Rankin scale scores; REND, rebound neurological deterioration.

The incidence of REND was significantly associated with lower rate of favorable functional outcomes in multivariable logistic regression analysis, after adjusting for age, baseline NIHSS, admission SBP, gender, TOAST, the presence of vessel occlusion or severe stenosis, the history of smoking and diabetes [adjusted OR (95%CI): 0.076 (0.031, 0.186), *P* < 0.001; Figure 2 and Table 2]. This association remained consistent after further adjustment for EVT (Supplementary Table S1). In addition, among patients with vessel occlusion or severe stenosis, those who developed REND had significantly lower reperfusion rates compared to those without REND (42.9% vs. 83.7%, *P* = 0.005). Stepwise logistic regression analysis identified that younger age [adjusted OR (95%CI): 0.966 (0.939, 0.994), *P* = 0.017], history of hypertension [adjusted OR (95%CI): 2.728 (1.165, 6.390), *P* = 0.021], and presence of large vessel occlusion/severe stenosis [adjusted OR (95%CI): 2.159 (1.076, 4.333), *P* = 0.030] were independent risk factors of REND (Table 3).

**TABLE 2:** The association between REND and favorable functional outcomes in multivariable logistic regression models.

Factors	OR (95%CI)	*P*-value
REND	0.076 (0.031, 0.186)	< 0.001
Age	0.956 (0.930, 0.983)	< 0.001
Baseline NIHSS	0.921 (0.878, 0.965)	< 0.001
Admission SBP	0.977 (0.964, 0.991)	< 0.001
Gender^#^	1.305 (0.633, 2.690)	0.471
Vessel occlusion or severe stenosis	0.570 (0.304, 1.070)	0.080
TOAST	1.101 (0.847, 1.432)	0.473
History of smoking	2.217 (1.110, 4.425)	0.024
History of diabetes	0.690 (0.348, 1.371)	0.289

**#**Using female as the reference. REND, rebound neurological deterioration; OR, odd ratio; NIHSS, National Institutes of Health Stroke Scale; SBP, systolic blood pressure.

**TABLE 3:** Independent predictors of REND in stepwise logistic regression analysis.

Factors	OR (95%CI)	*P*-value
Age	0.966 (0.939, 0.994)	0.017
Hypertension	2.728 (1.165, 6.390)	0.021
Vessel occlusion or severe stenosis	2.159 (1.076, 4.333)	0.030

Age, hypertension, vessel occlusion or severe stenosis, NLR, fibrinogen and DDI were included in stepwise logistic regression model. REND, rebound neurological deterioration; OR, odd ratio; NLR, neutrophil-lymphocyte ratio; DDI, D-dimer.

The risk of poor prognosis was further amplified in patients with substantial REND, with only 11.1% achieving favorable functional outcomes at 90 days (Supplementary Figure S1). The comparison of demographic, imaging, and clinical characteristics between patients with and without substantial REND was showed in Supplementary Table S2. The stepwise logistic regression analysis revealed that younger age [adjusted OR (95%CI): 0.958 (0.927, 0.989), *P* = 0.009], higher systolic blood pressure (SBP) at admission [adjusted OR (95%CI):1.018 (1.000, 1.036), *P* = 0.045] and vessel occlusion or severe stenosis [2.677 (1.129, 6.345), *P* = 0.025] were independent risk factors of substantial REND (Table 4).

**TABLE 4:** Independent predictors of substantial REND in stepwise logistic regression analysis.

Factors	OR (95%CI)	*P*-value
Age	0.958 (0.927, 0.989)	0.009
Admission SBP	1.018 (1.000, 1.036)	0.045
Vessel occlusion or severe stenosis	2.677 (1.129, 6.345)	0.025

Age, hypertension, vessel occlusion or severe stenosis, NLR and DDI were included in stepwise logistic regression model. REND, rebound neurological deterioration; OR, odd ratio; SBP, systolic blood pressure; NLR, neutrophil-lymphocyte ratio; DDI, D-dimer.

## Discussion

In this study, we found that REND was observed in approximately 20% of patients who initially achieved ENI following IVT and was significantly associated with unfavorable functional outcomes at 90 days. Moreover, younger age, history of hypertension, and the presence of large vessel occlusion or severe stenosis were identified as independent risk factors of REND.

To our knowledge, this is the first study to characterize the “improvement-then-deterioration” trajectory following IVT and to propose the concept of REND. Although ENI occurring within 2 h of IVT initiation has been demonstrated to significantly increase the likelihood of favorable functional outcomes at 90 days (Hemmen et al., 2011; Kharitonova et al., 2011) and is often regarded as a marker of clinical stability assuring less intensive monitoring (Anderson et al., 2025), our findings revealed that 1/5 patients who initially demonstrated ENI would experience symptom fluctuations and were less likely to achieve good prognosis. These results underscored the importance of recognizing REND as a distinct clinical trajectory, given that interim deterioration may reflect ongoing pathological processes and contribute to poor functional outcomes.

To better understand the potential mechanisms and facilitate early identification, we further explored clinical and imaging predictors of REND. In our study, history of hypertension emerged as an independent predictor of REND and elevated SBP at admission was significantly associated with substantial REND, consistent with previous studies on END following reperfusion therapy (Liu et al., 2023; Sabir Rashid et al., 2022; Shi et al., 2023). We hypothesized that blood pressure might play a pivotal role in the development of REND by altering cerebral hemodynamic reserve. Chronic hypertension and elevated admission SBP have been demonstrated to impair cerebral autoregulation and induce vascular remodeling (Santisteban et al., 2023; van den Kerkhof et al., 2023), ultimately leading to reduced global cerebral blood flow (CBF) (Muller et al., 2012). Our prior study further showed that increased baseline BP was associated with reduced CBF in the contralateral hemisphere of acute small subcortical infarcts (He et al., 2024), reflecting exhausted compensatory capacity (Rudilosso et al., 2019). These hemodynamic vulnerabilities may contribute to secondary deterioration, despite an initial favorable response to IVT. Beyond hemodynamic alterations, chronic hypertension may compromise blood-brain barrier (BBB) integrity through sustained endothelial injury (Chen et al., 2025). Although reperfusion after IVT may initially restore cerebral blood flow and result in ENI, BBB disruption may facilitate inflammatory and oxidative injury (Candelario-Jalil et al., 2022), leading to vasogenic edema and delayed neurological worsening. In addition, chronic hypertension is associated with endothelial dysfunction (Santisteban et al., 2020), which may facilitate microvascular platelet aggregation and thrombosis (Schäfer and Bauersachs, 2008) after recanalization, leading to recurrent perfusion impairment despite initial clinical improvement. However, whether antihypertensive therapy mitigates the risk of REND remains unclear and further studies are needed to determine optimal BP targets in this population, especially given evidence that robust BP reduction may exacerbate focal hypoperfusion and promote END (Vynckier et al., 2021; Werring et al., 2025).

Although ENI within 2 h after initiation of IVT is commonly regarded as a surrogate marker for early successful recanalization (Kharitonova et al., 2011) and a favorable sign that may prevent additional endovascular treatment among patients with vessel occlusion or severe stenosis in clinical practice, this single-center cohort showed that part of patients with vessel occlusion and severe stenosis failed to achieve reperfusion despite experiencing ENI, suggesting that the conventional definition of ENI may not reliably predict successful recanalization following IVT (Che et al., 2024). Moreover, our results revealed that large vessel occlusion or severe stenosis was independently associated with REND and patients with REND were less likely to achieve successful reperfusion on follow-up CTP compared to those without REND. However, no significant difference in reperfusion rates was observed between patients with and without substantial REND, which might be attributed to the limited sample size, as only 8 patients with substantial REND underwent follow-up CTP imaging. We hypothesized that transient clinical improvement in patients following IVT might be driven by fragile collateral circulation (Broocks et al., 2023) rather than successful recanalization and persistent vessel occlusion or severe stenosis would lead to subsequent collateral exhaustion and symptom fluctuations (Conrad et al., 2020; Seners and Baron, 2018; Seners et al., 2014; Tisserand et al., 2014). In addition, incomplete or unstable recanalization after IVT in patient with vessel occlusion or severe stenosis might increase the risk of re-occlusion or distal embolization, both of which could impair downstream perfusion and result in delayed clinical worsening (Bala et al., 2023; Seners and Baron, 2018). These findings highlighted the potential value of early digital subtraction angiography (DSA) in assessing vascular status and guiding timely endovascular treatment in patients with vessel occlusion or severe stenosis who initially exhibit ENI following IVT.

Previous studies have shown that older age was associated with a higher risk of END (Liu et al., 2023; Tian et al., 2023). Interestingly, our study found that younger patients were more likely to experience REND, which could be partially explained by the higher prevalence of atrial fibrillation among older patients. Cardiogenic clots are typically richer in fibrin and have a looser structure, which may allow better penetration of thrombolytic agents and facilitate reperfusion (Jolugbo and Ariens, 2021). In addition, intracranial arterial dissection is more prevalent in younger patients and may partially contribute to the association between younger age and REND (Ekker et al., 2018). However, there was no significant difference in TOAST classification between patients with and without REND in our cohort, which may be attributed to the limited sample size and needs further investigation. Moreover, age-related differences in inflammatory and immune responses to ischemia and reperfusion injury may contribute to variability in REND risk, as aging is associated with altered microglial transcriptional profiles and immune reactivity (Candelario-Jalil et al., 2022; Finger et al., 2022). Consistent with this hypothesis, patients with REND in our cohort exhibited a significantly higher baseline NLR, suggesting a heightened systemic inflammatory state.

In summary, the strength of our study is that we propose and systematically characterize REND as a distinct “improvement–then–deterioration” trajectory after IVT, separate from END, but comparably associated with unfavorable functional outcomes in AIS patients. Our findings emphasized that initial ENI did not necessarily translate into favorable functional outcomes. REND occurred in approximately one-fifth of patients who exhibited ENI following IVT and was associated with unfavorable functional outcomes, highlighting the importance of continued neurological monitoring and timely interventions in patients who initially respond well to IVT. Younger age, history of hypertension, and the presence of large vessel occlusion or severe stenosis were significantly associated with REND, providing practical value for risk stratification and treatment decisions after IVT. The Safety and Efficacy of Tirofiban for the Prevention of Neurological Deterioration in Acute Ischemic Stroke (TREND) (Zhao et al., 2024) and Advancing Stroke Safety and Efficacy through Early Tirofiban Administration after Intravenous Thrombolysis (ASSET-IT) (Tao et al., 2025) trials have demonstrated that intravenous tirofiban could reduce the incidence of END and administration of tirofiban within 1 h after IVT increased the likelihood of favorable functional outcomes. The potential role of tirofiban in preventing REND should be specifically evaluated in future studies. In addition, The Early antiplatelet for minor stroke following thrombolysis (EAST) trial (NCT05193071) has showed that early antiplatelet treatment with clopidogrel plus aspirin did not reduce the risk of END nor improve excellent functional outcome at 90 days among patients with minor stroke after IVT (Chen et al., 2026; Li et al., 2023). Whether early antiplatelet therapy may help prevent the occurrence of REND, as well as the optimal timing and dosage, warrants further investigation.

Several limitations should be taken into account. Firstly, the assessment of NIHSS scores is subject to interrater variability, which may affect the accuracy of REND classification. To mitigate this limitation, we additionally defined “substantial REND” using a more stringent threshold to reduce subjective inconsistencies and improve the robustness of our findings. Secondly, the pathophysiological mechanisms underlying neurological deterioration may differ by stroke subtype. However, patients in our study were not stratified by stroke etiology, and the potential influence of infarct patterns and location on the occurrence of REND was not specifically investigated due to the limited sample size. Thirdly, the absence of serial imaging and NIHSS score assessments at multiple time points following IVT limited our ability to delineate the dynamic evolution and underlying mechanisms of REND, which should be further explored in the future. In addition, the single-center design may limit the generalizability of our findings, which required confirmation and extension in larger and multicenter cohorts.

## Conclusion

In this single-center cohort study, REND occurred in approximately 20% of patients who exhibited ENI following IVT and was associated with unfavorable functional outcomes. Younger age, a history of hypertension, and the presence of large vessel occlusion or severe stenosis were independently associated with an increased risk of REND.

## Data Availability

The raw data supporting the conclusions of this article will be made available by the authors, without undue reservation.
