# Supplementary material for: Incidence, outcomes and risk factors of rebound neurological deterioration after initial improvement following intravenous thrombolysis: a retrospective cohort study
Source: Front Aging Neurosci. 2026 Mar 27;18:1706795. doi: 10.3389/fnagi.2026.1706795 (PMC13066128; doi:10.3389/fnagi.2026.1706795)
Supplement: Supplementary file 1 [file Data_Sheet_1.docx]

**Table S1: The association between REND and favorable functional outcomes after including EVT in multivariable logistic regression models**

|  | **OR (95%CI)** | **P-value** |
| --- | --- | --- |
| REND | 0.078 (0.032, 0.192) | <0.001 |
| Age | 0.955 (0.928, 0.982) | 0.001 |
| Baseline NIHSS | 0.917 (0.872, 0.964) | 0.001 |
| Admission SBP | 0.978 (0.964, 0.991) | 0.001 |
| EVT | 1.223 (0.462, 3.237) | 0.686 |
| Gender^#^ | 1.255 (0.603, 2.611) | 0.543 |
| Vessel occlusion or severe stenosis | 0.515 (0.265, 1.000) | 0.050 |
| TOAST | 1.132 (0.868, 1.478) | 0.360 |
| History of smoking | 2.313 (1.146, 4.669) | 0.019 |
| History of diabetes | 0.703 (0.351, 1.407) | 0.320 |

**#** using female as the reference

Abbreviation: REND: rebound neurological deterioration; OR: odd ratio; NIHSS: National Institutes of Health Stroke Scale; SBP: systolic blood pressure; EVT: endovascular treatment

**Table S2: The comparison of demographic, imaging, and clinical characteristics between patient with and without substantial REND**

|  | **Without substantial REND**  **(n = 241)** | **Substantial REND**  **(n = 36)** | **P-Value** |
| --- | --- | --- | --- |
| **Demographics** |  |  |  |
| Male sex, n (%) | 175 (72.6%) | 24 (66.7%) | 0.551 |
| Age, years, median (IQR) | 69 (60, 76) | 61 (55, 73) | 0.011 |
| **Medical history** |  |  |  |
| Smoking, n (%) | 107 (44.4%) | 15 (41.7%) | 0.858 |
| Hypertension, n (%) | 157 (65.1%) | 27 (75%) | 0.264 |
| Diabetes mellitus, n (%) | 58 (24.1%) | 11 (30.6%) | 0.412 |
| Hyperlipidemia, n (%) | 35 (14.5%) | 2 (5.6%) | 0.190 |
| Stroke, n (%) | 49 (20.3%) | 6 (16.7%) | 0.664 |
| Atrial fibrillation, n (%) | 52 (21.6%) | 6 (16.7%) | 0.526 |
| **Clinical features** |  |  |  |
| Admission NIHSS, median (IQR) | 10 (7, 16) | 11 (8, 14) | 0.543 |
| NIHSS_2h，median (IQR) | 3 (1, 7) | 4 (1, 6) | 0.959 |
| NIHSS_24h, median (IQR) | 2 (0, 5) | 11 (7, 13) | <0.001 |
| OTD, min, median (IQR) | 94 (52, 152) | 95 (66, 127) | 0.886 |
| OTT, min, median (IQR) | 154 (102, 215) | 157 (104, 197) | 0.836 |
| Admission SBP, mmHg, median (IQR) | 147 (132, 166) | 158 (136, 175) | 0.049 |
| Admission DBP, mmHg, median (IQR) | 83 (76, 92) | 89 (77, 98) | 0.111 |
| Plasma glucose, mmol/L, median (IQR) | 6.9 (5.7, 9.2) | 6.8 (5.8, 9.6) | 0.902 |
| Baseline NLR, median (IQR) | 3.20 (1.94, 5.36) | 4.70 (3.06, 8.13) | 0.004 |
| Baseline FIB, g/L, median (IQR) | 2.7 (2.3, 3.3) | 2.8 (2.6, 3.4) | 0.126 |
| Baseline DDI, mg/L FEU, median (IQR) | 0.67(0.32, 1.84) | 1.51(0.42, 2.76) | 0.074 |
| EVT, n (%) | 32 (14%) | 5 (14.7%) | 1.000 |
| Vessel occlusion or severe stenosis, n (%) | 107 (44.4%) | 23 (63.9%) | 0.032 |
| **Site, n (%)** |  |  | 0.043 |
| ICA | 22 (9.1%) | 7 (19.4%) |  |
| Tandem | 2 (0.8%) | 0 (0%) |  |
| MCA | 67 (27.8%) | 11(30.5%) |  |
| ACA | 6 (2.5%) | 0 (0%) |  |
| PCA | 2 (0.8%) | 0 (0%) |  |
| BA | 8 (3.33%) | 4 (11.1%) |  |
| VA | 0 (0%) | 1 (2.8%) |  |
| **Brain Perfusion Imaging Features** |  |  |  |
| Reperfusion, n (%) * | 37 (75.5%) | 5 (62.5%) | 0.422 |
| **TOAST, n (%)** |  |  | 0.663 |
| Large artery atherosclerosis | 79 (32.8%) | 15 (41.7%) |  |
| Cardioembolism | 56 (23.2%) | 6 (16.7%) |  |
| Small vessel occlusion | 50 (20.7%) | 6 (16.7%) |  |
| Other/Undetermined cause | 56 (23.2%) | 9 (25%) |  |
| **Prognosis** |  |  |  |
| Favorable functional outcomes, n (%) | 151 (62.7%) | 4 (11.1%) | <0.001 |

Abbreviation: REND: rebound neurological deterioration; NIHSS: National Institutes of Health Stroke Scale; OTD: onset to door; OTT: onset to treatment; SBP: systolic blood pressure; DBP: diastolic blood pressure; NLR: neutrophil – lymphocyte ratio; FIB: fibrinogen; DDI: D-dimer; EVT: endovascular treatment; ICA: internal carotid artery; MCA: middle cerebral artery; ACA: anterior cerebral artery; PCA: posterior cerebral artery; BA: basilar artery; VA: vertebral artery;

*Among 57 patients who completed both baseline and follow-up CTP imaging, 8 had substantial REND.

**Figure S1:** **Distribution of Modified Rankin Scale Scores at 90 Days in patients with and without substantial REND**


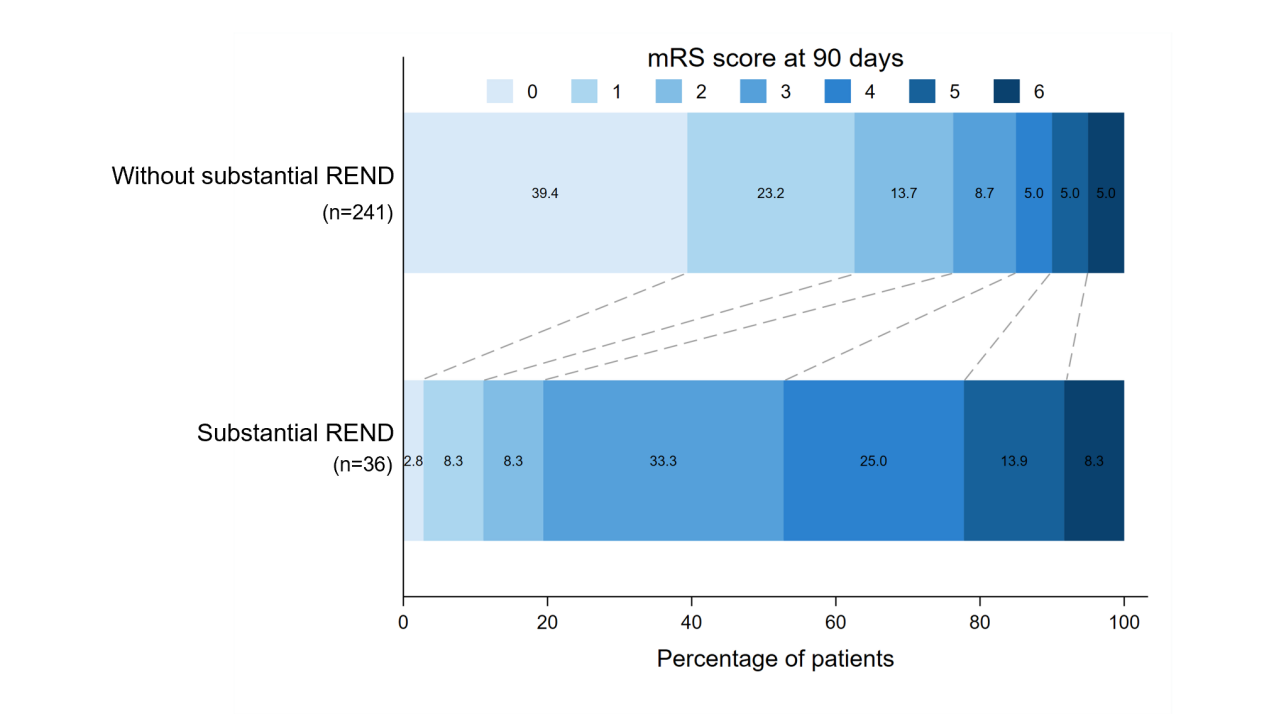


Abbreviations: mRS: modified Rankin scale scores; REND: rebound neurological deterioration
